# Construction and validation a nomogram to predict overall survival for colorectal signet ring cell carcinoma

**DOI:** 10.1038/s41598-021-82978-8

**Published:** 2021-02-09

**Authors:** Jian-dong Diao, Li-xia Ma, Chun-jiao Wu, Xian-hong Liu, Xiao-yun Su, Hong-yu Bi, Bo Bao, Hao-wei Yan, Lei Shi, Yong-jing Yang

**Affiliations:** 1 Department of Oncology and Hematology, China-Japan Union Hospital of Jilin University, Changchun, Jilin 130033 China; 2 Departments of Oncology, Jilin Cancer Hospital, Changchun, Jilin 130012 China; 3 School of Pharmaceutical Sciences, Jilin University, Changchun, Jilin 130012 China; 4 Department of Radiation Oncology, Jilin Cancer Hospital, Changchun, Jilin 130012 China; 5 Department of Regeneratve Medicine, School of Pharmacy, Jilin University, Changchun, Jilin 130012 China; 6 Prevention and Health Care Department, Jilin Cancer Hospital, Changchun, Jilin 130012 China

**Keywords:** Cancer, Oncology, Risk factors

## Abstract

To construct and validate a nomogram to predict the overall survival (OS) of colorectal signet ring cell carcinoma (SRCC). The potentially eligible cases were obtained against the SEER database from 2004 to 2015. Log-rank test and Cox analysis were conducted to identify the independent prognostic factors for predicting OS. The identified prognostic factors were later integrated for the construction of an OS prediction nomogram. Altogether 2904 eligible cases were identified, and the median survival time was 18 (range: 0–155) months. As suggested by multivariate analysis, age, primary site, grade, tumor size, T stage, N stage, M stage, surgery, lymph node dissection and chemotherapy were identified as the independent factors for predicting OS. Afterwards, the above variables were incorporated into the nomogram. The C-index indicated better discriminatory ability of the nomogram than AJCC 8th TNM staging and SEER summary stage systems (both *P* < 0.001). Calibration plots further showed good consistency between the nomogram prediction and actual observation. The time independent area under the curves (tAUCs) for 3-year and 5-year OS in nomogram were larger than AJCC and SEER summary stage system. The constructed nomogram could potentially predict the survival of colorectal SRCC individuals.

## Introduction

Colorectal cancer (CRC) is the second most common cause of cancer-associated mortality in the United States, and it has posed a great threat to global health^1^. In recent years, colorectal signet ring cell carcinoma (SRCC), one of the CRC subtypes^2^, has aroused wide attention. It is extensively reported that, SRCC commonly originates from the undifferentiated colorectal mucosal stem cells; therefore, fast proliferation, low differentiation level, metastasis and diffuse infiltration can be frequently detected^3,4^. In addition, SRCC is also identified by the AJCC 7th TNM classification system as the independent factor to predict the adverse prognosis^5^. Nonetheless, there are only small case series and case reports available for colorectal SRCC, while information regarding its clinicopathological characteristics and prognostic outcomes remains largely unexplored^6^. In this regard, it is important to precisely estimate the prognosis of SRCC cases, which may facilitate the development of risk-based individualized treatment and the best therapeutic strategies.

TNM stage system is a prevalent method to predict the outcomes in tumor patients through assessing tumor size and location (T), regional lymph node metastasis (N) and distant metastasis (M)^7^. However, TNM classification is not efficient to encompass cancer biology as well as to precisely predict outcomes of colorectal SRCC^8^. In addition, other clinicopathological parameters can also affect prognosis in SRCC patients, including tumor grade, tumor site, race, age and therapy^4,9^. Hence, it is urgently demanded to establish a novel stage classification involving tumor features and patient status.

As a simple, user-friendly statistical method, nomogram has been uncovered to harbor comparative or even superior predictive capacity over conventional TNM stage systems in various types of malignancies^10–12^. To be specific, the successful establishment of nomogram should not only consider the prognostic weight of every parameter to calculate the possibility of an outcome but incorporate several independent indicators for optimal conclusion. Of note, nomograms are capable of accurately estimating survival for individuals via the assessment of vital prognostic factors than TNM stage system^13,14^. However, as far as we know, there is no such population-based nomogram specifically for colorectal SRCC. To this end, we aimed at constructing and verifying a nomogram for OS prediction in colorectal SRCC based on Surveillance, Epidemiology and End Results (SEER) database.

## Materials and methods

### Ethics statement

SEER is the greatest cancer database with the highest authoritativeness in North America^15^, which includes cancer data through covering nearly 30% of the US populations across different geographic regions that can stand for population diversity^16^. To collect related information from this database, the SEER Research Data Agreement (No. 19817-Nov2018) was signed in this study, and data were searched against this database in line with those approved guidelines. The extracted data were publicly accessible and de-identified, and the data analysis was considered as non-human subjects by Office for Human Research Protection, thus, no approval was demanded from institutional review board.

### Study population

The eligible cases were screened using the SEER*State v8.3.6 approach (released on August 8th, 2019). In the present work, we included 18 SEER regions between 2004 and 2015. Patients conforming to the following criteria were enrolled: (1) those with primary colorectal SRCC; and (2) those with SRCC diagnosed according to the third version of the International Classification of Disease for Oncology (ICD-O-3; coded as 8490/3). At the same time, patients conforming to any one of the following conditions were excluded from this study: (1) patients with more than one primary tumor; (2) those with clinical diagnosis, or those diagnosed based on autopsy or the death certificate; (3) those with insufficient data like the mode of surgery and AJCC stage; (4) those whose tumor location was not mentioned; (5) those with unavailable information on prognosis. The remaining participants were included into the initial SEER cohort. For establishing and validating the nomogram, the enrolled cases were randomized into the training or validation set.

### Covariates and endpoint

The following patient characteristics were analyzed, including gender, age,race, marital status, insurance status, primary site, year of diagnosis, tumor size, grade,T, N, M stage, surgery, lymph node dissection, radiotherapy and chemotherapy. In this study, the single (never married), widowed (having a domestic partner), separated and divorced cases were classified into unmarried category^17^. With regard to the primary tumor site, it was divided into cecum–transverse colon (such as appendix, cecum, ascending colon, transverse colon, hepatic flexure), descending colon–sigmoid (like descending colon, sigmoid colon, splenic flexure), multiple, rectum and unknown^18^. As for the year of diagnosis, it was classified as 2004–2007, 2008–2011, 2012–2015, in line with previous studies^19,20^. At the same time, tumor size and age were grouped according to previous articles as well^21–24^. The cancer stage was classified according to the AJCC 6th classification system that was adapted to SEER-derived patients diagnosed from 2004 to 2015. Meanwhile, the qualified patients were further regrouped in line with the AJCC 8th TNM classification system. In this study, the endpoint was set as overall survival (OS), which referred to the duration between diagnosis and death due to all causes^25^.

### Statistical analysis

#### Nomogram construction

Categorical variables were compared by Fisher's exact test or chi-square test and expressed in the manner of proportions and frequencies. Univariate analysis was conducted to predict prognosis using the Kaplan–Meier (K–M) approach as well as log-rank test. Upon univariate analysis, variables with *P-*value ≤ 0.1 were screened for multivariate backward stepwise Cox proportional hazard analysis to determine each possible independent risk factor. Additionally, multicollinearity diagnostics in statistical modeling were conducted by evaluating the correlations, variance inflation factors, and eigenvalues. Then, we established a nomogram model (based on identified prognostic factors) to predict 3-year, 5-year OS in the training and validation cohort using the rms package of R. We determined the total nomogram score for every case based on every variable score in the contour diagram for modeling group.

#### Nomogram validation

The nomogram was validated by determining its discrimination and calibration abilities using the internal (training) as well as external (validation) set, respectively. In addition, we used the concordance index (C-index) for evaluating our model discrimination performance and assessing the difference in the predicting ability between predicted and observed values^26^. As a result, the higher C-index value indicated the better patient discrimination ability among different prognostic outcomes. Also, we used the Rcorrp.cens package of Hmisc in R software for comparing the different results from the existing 8th TNM classification system and the SEER summary stage system, and utilized C-index to determine them. Using the marginal estimate versus model, plots presenting the calibration of predicted with measured survival outcomes were made, where the 45-degree plot was the optimal model with marked consistency in terms of the outcomes. The Receiver Operating Characteristic (ROC) curves were also plotted to validate the nomogram score. In this study, the bootstrapping re-sampling approach (1000 repetitions) was applied in obtaining the comparatively unbiased estimates and checking interval validation. Statistical analysis was conducted using SPSS19.0 (SPSS Inc., Chicago, USA) and R (version 3.51, www.r-project.org). A difference of *P* < 0.05 (two-tailed) was deemed to be statistically significant.

## Results

### Patient characteristics

**Figure 1:**
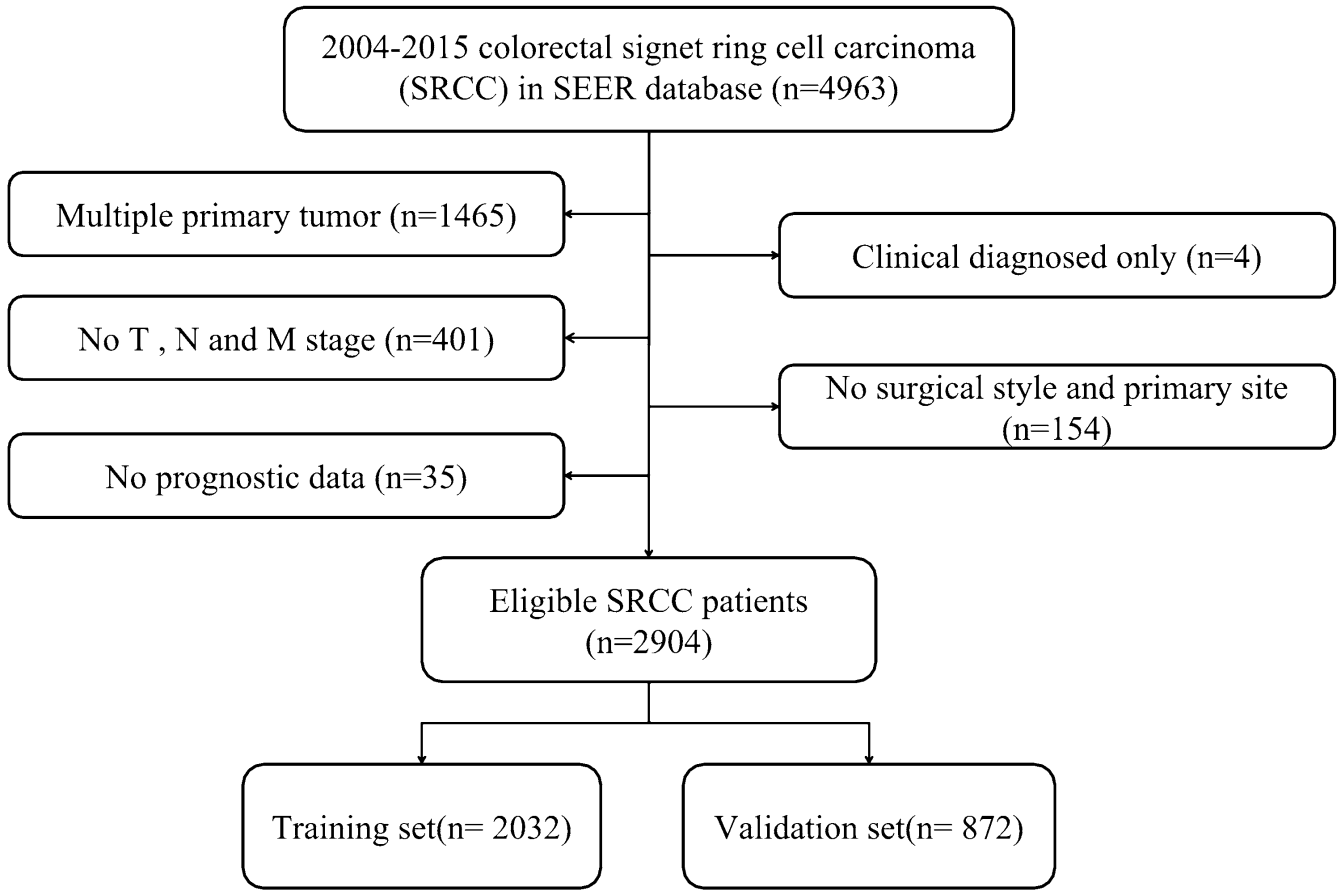
Flow chart for patients selection.

In total, 2904 qualified subjects who were diagnosed with colorectal SRCC from 2004 to 2015 were enrolled in this research. In addition, 2032 and 872 subjects were assigned into the training and validation cohorts, respectively. The flow chart of data selection was displayed in Fig. 1. Among all the subjects, 50.69% were males, with a median age was 63 years (range: 12–103 years). Most subjects were married (53.99%) and white (81.61%). Cecum–transverse colon (61.95%) was the most prevalent tumor site, followed by rectum (19.42%), descending colon–sigmoid (15.87%) and multiple (1.48%). Tumor size ˃ 5 cm (42.84%) was the most common. Most cases of colorectal SRCC had advanced clinical stage (T3: 44.42%; T4:45.83%; N2:46.83%) and advanced pathological grade (grade III/IV: 80.65%).Operations were performed on 2568 (88.43%) patients, of which 1728 (59.50%) were total colectomy/ proctectomy. Most patients (79.72%) had more than four lymph nodes removed. More than half of the patients received chemotherapy (58.13%) and only 13.83% patients received radiotherapy. The median survival time was 18.0 months (0–155 months). The 3- and 5-year OS rates were 35.6%, and 28.1%. The demographic and clinicopathological features were listed in Table 1, indicating no significant difference between two groups.

**Table 1:** Patient demographics and pathological characteristics.

Variables	All patients N (%)	Validation set N (%)	Training set N (%)	*P* value
Year at diagnosis				0.479
2004–2007	984 (33.88)	292 (33.49)	692 (34.06)	
2008–2011	954 (32.85)	300 (34.40)	654 (32.19)	
2012–2015	966 (33.26)	280 (32.11)	686 (33.76)	
Insured status				0.736
Uninsured/unknown	903 (31.10)	275 (31.54)	628 (30.91)	
Any medicaid/insured	2001 (68.90)	597 (68.46)	1404 (69.09)	
Age				0.528
˂65	1516 (52.20)	463 (53.10)	1053 (51.82)	
≥ 65	1388 (47.80)	409 (46.90)	979 (48.18)	
Marital status				0.924
Unmarried	1336 (46.01)	400 (45.87)	936 (46.06)	
Married	1568 (53.99)	472 (54.13)	1096 (53.94)	
Gender				0.373
Female	1432 (49.31)	441 (50.57)	991 (48.77)	
Male	1472 (50.69)	431 (49.43)	1041 (51.23)	
Race				0.327
Black	275 (9.47)	79 (9.06)	196 (9.65)	
White	2370 (81.61)	705 (80.85)	1665 (81.94)	
Other	259 (8.92)	88 (10.09)	171 (8.42)	
Primary site				0.510
Cecum–transverse colon	1799 (61.95)	527 (60.44)	1272 (62.60)	
Descending colon–sigmoid	461 (15.87)	145 (16.63)	316 (15.55)	
Multiple	43 (1.48)	10 (1.15)	33 (1.62)	
Rectum	564 (19.42)	176 (20.18)	388 (19.09)	
Unknown	37 (1.27)	14 (1.61)	23 (1.13)	
Grade				0.155
Grade I/II	173 (5.96)	63 (7.22)	110 (5.41)	
Grade III/IV	2342 (80.65)	697 (79.93)	1645 (80.95)	
Unknown	389 (13.40)	112 (12.84)	277 (13.63)	
Tumor size				0.882
≤ 5 cm	1179 (40.60)	360 (41.28)	819 (40.31)	
˃5 cm	1244 (42.84)	370 (42.43)	874 (43.01)	
Unknown	481 (16.56)	142 (16.28)	339 (16.68)	
T stage				0.079
T0-Tis	16 (0.55)	4 (0.46)	12 (0.59)	
T1	167 (5.75)	61 (7.00)	106 (5.22)	
T2	100 (3.44)	35 (4.01)	65 (3.20)	
T3	1290 (44.42)	401 (45.99)	889 (43.75)	
T4	1331 (45.83)	371 (42.55)	960 (47.24)	
N stage				0.269
N0	854 (29.41)	274 (31.42)	580 (28.54)	
N1	690 (23.76)	197 (22.59)	493 (24.26)	
N2	1360 (46.83)	401 (45.99)	959 (47.19)	
M stage				0.713
M0	1827 (62.91)	553 (63.42)	1274 (62.70)	
M1	1077 (37.09)	319 (36.58)	758 (37.30)	
Surgery				0.841
No surgery	336 (11.57)	104 (11.93)	232 (11.42)	
Local tumor excision/partial colectomy	840 (28.93)	256 (29.36)	584 (28.74)	
Total colectomy	1728 (59.50)	512 (58.72)	1216 (59.84)	
Lymph node dissection				0.717
None or biopsy	507 (17.46)	148 (16.97)	359 (17.67)	
1 to 3	82 (2.82)	22 (2.52)	60 (2.95)	
≥ 4	2315 (79.72)	702 (80.50)	1613 (79.38)	
Chemotherapy				0.418
No/unknown	1216 (41.87)	375 (43.00)	841 (41.39)	
Yes	1688 (58.13)	497 (57.00)	1191 (58.61)	
Radiotherapy				0.751
No/unknown	2502 (86.16)	754 (86.47)	1748 (86.02)	
Yes	402 (13.84)	118 (13.53)	284 (13.98)	

### Nomogram construction

**Figure 2:**
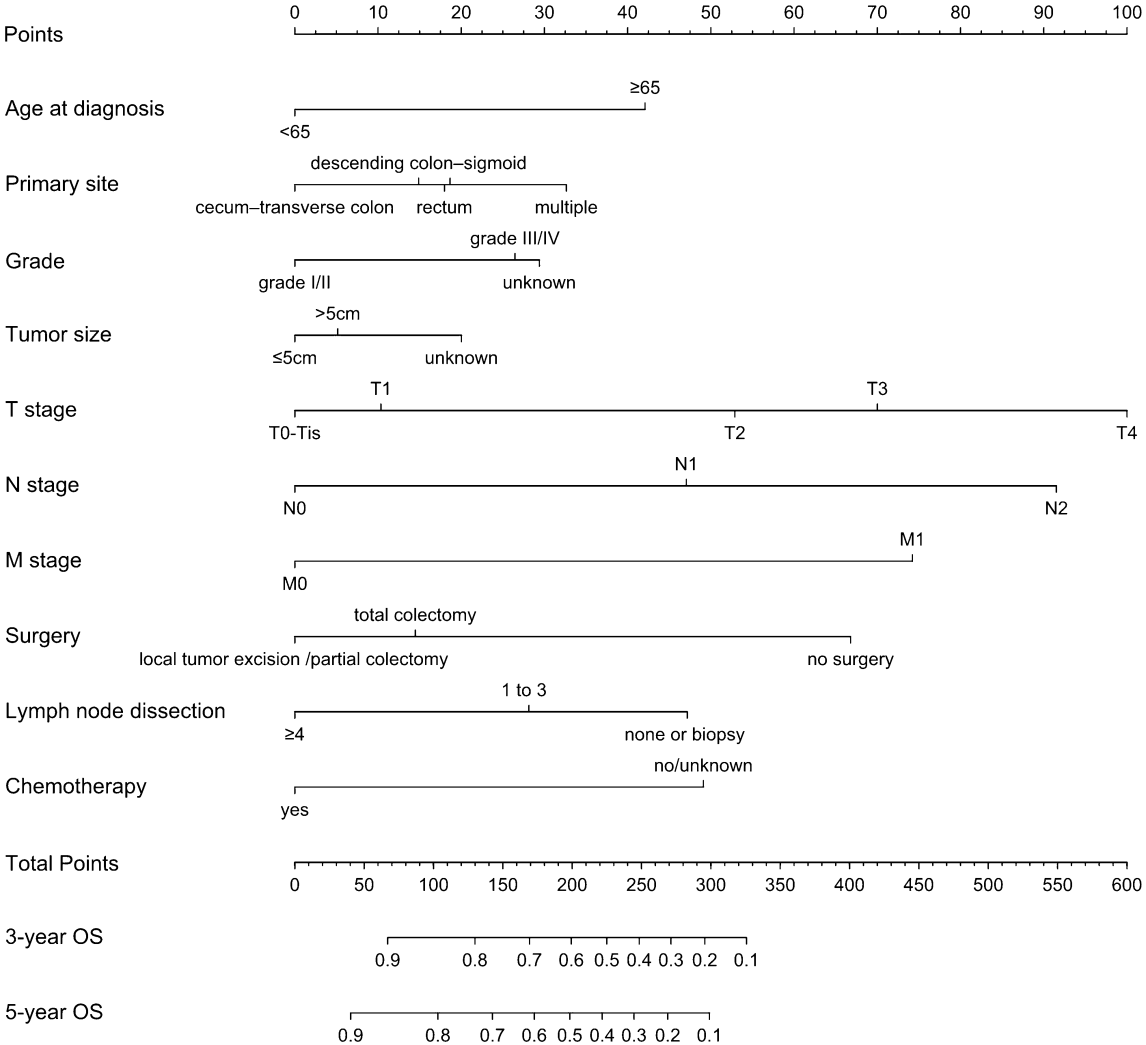
Nomogram to predict 3-year (**A**) and 5-year (**B**) overall survival (OS) of colorectal SRCC patients.

Univariate analysis revealed 11 indicators could affect OS (shown in Table 2). Among them, marital status was included as an adjusted variable in the step-wise modeling. Consequently, multivariate analysis showed that age, primary site, grade, tumor size, T stage, N stage, M stage, surgery, lymph node dissection and chemotherapy were independent predictive indicators of OS (all *P* < 0.05). Multicollinearity diagnostic tests including pair-wise correlations, variance inflation factors plot and eigenvalues plot suggested that severe multicollinearity issues would not exist (Supplemental Figs. 1 and 2). A nomogram for 3- and 5-year OS prediction was constructed according to these independent factors (Fig. 2). Nomogram uncovered that AJCC stage made the greatest contribution to prognosis, followed by surgery, chemotherapy, number of lymph node dissection and age. By adding the scores of each selected variable, the likelihood of survival of the individual patient can be easily calculated.

**Table 2:** Univariate and multivariate analyses of overall survival (OS) for patients in training set.

Variable	Univariate analysis	Multivariate analysis	
	*P* value	HR (95% CI)	*P* value
Year at diagnosis	0.801		NI
2004–2007			
2008–2011			
2012–2015			
Insured status	0.532		NI
Uninsured/unknown			
Any medicaid/insured			
Age	0.001		
˂65		Reference	
≥ 65		1.622 (1.452, 1.813)	˂0.001
Marital status^a^	< 0.001		
Unmarried		Reference	
Married		0.869 (0.783, 0.964)	0.008
Gender	0.842		NI
Female			
Male			
Race	0.143		NI
Black			
White			
Other			
Primary site	< 0.001		
Cecum–transverse colon		Reference	
Descending colon–sigmoid		1.188 (1.020, 1.385)	0.027
Multiple		1.406 (0.967, 2.044)	0.074
Rectum		1.227 (1.055, 1.434)	0.010
Unknown		1.237 (0.775, 1.974)	0.372
Grade	< 0.001		
Grade I/II		Reference	
Grade III/IV		1.364 (1.061, 1.753)	0.015
Unknown		1.407 (1.060, 1.868)	0.018
Tumor size	< 0.001		
≤ 5 cm		Reference	
˃5 cm		0.950 (0.845, 1.067)	0.387
Unknown		1.197 (1.016, 1.411)	0.032
T stage	< 0.001		
T0-Tis		Reference	
T1		1.134 (0.387, 3.324)	0.819
T2		1.881 (0.679, 5.209)	0.224
T3		2.330 (0.861, 6.304)	0.096
T4		3.306 (1.221, 8.952)	0.019
N stage	< 0.001		< 0.001
N0		Reference	
N1		1.716 (1.457, 2.021)	< 0.001
N2		2.898 (2.479, 3.388)	< 0.001
M stage	< 0.001		
M0		Reference	
M1		2.377 (2.104, 2.685)	< 0.001
Surgery	< 0.001		< 0.001
No surgery		Reference	
Local tumor excision/partial colectomy		0.468 (0.365, 0.599)	< 0.001
Total colectomy		0.552 (0.429, 0.712)	< 0.001
Lymph node dissection	< 0.001		< 0.001
None or Biopsy		Reference	
1 to 3		0.782 (0.561, 1.091)	0.148
≥ 4		0.573 (0.462, 0.710)	< 0.001
Chemotherapy	0.002		
No/unknown		Reference	
Yes		0.583 (0.519, 0.654)	< 0.001
Radiotherapy	0.280		
No/unknown			
Yes			

*NI* not included in the multivariate survival analysis, ^a^marital status was used as an adjusted factor in multivariate analysis.

### Nomogram validation

**Figure 3:**
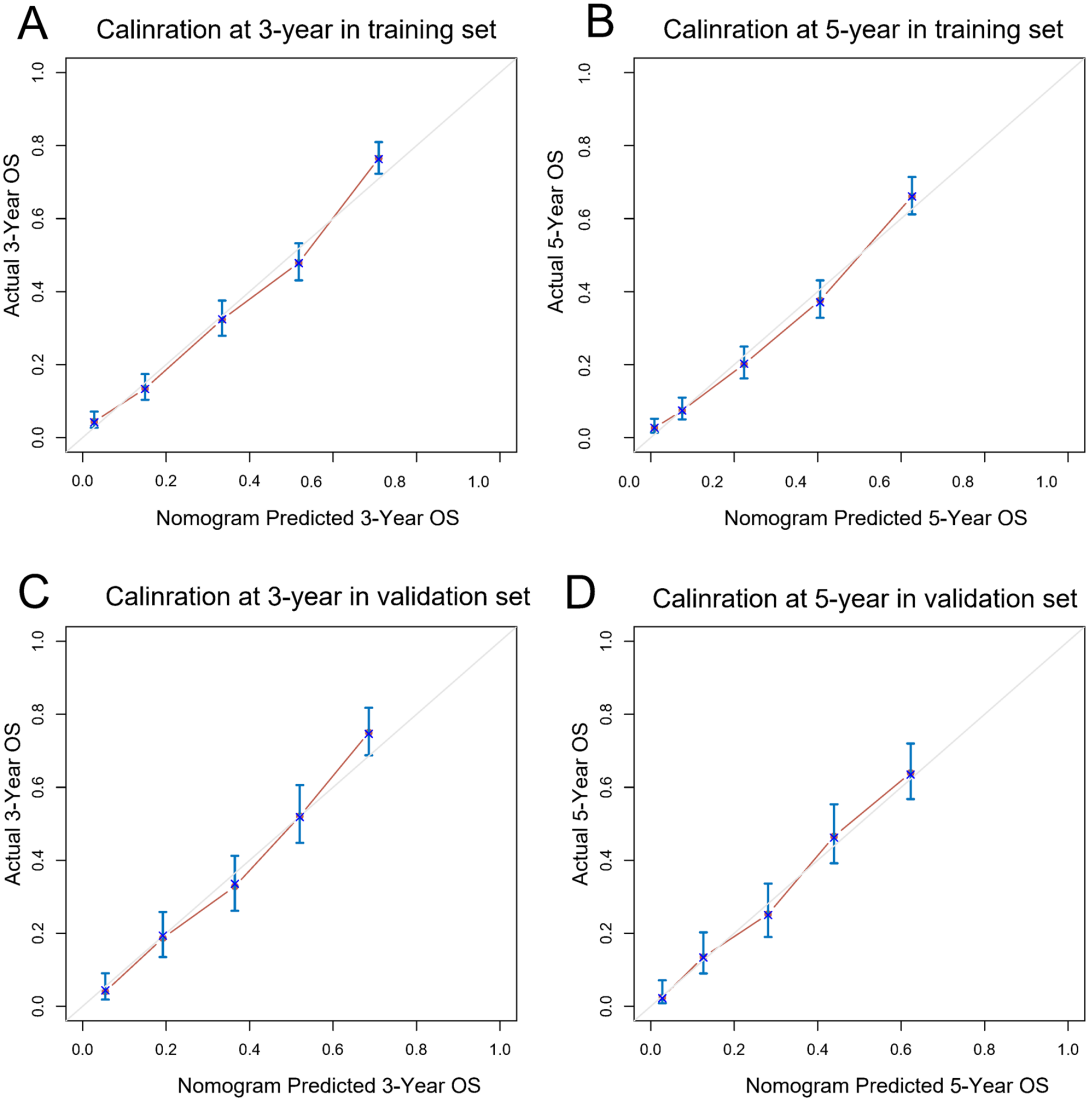
Calibration plots of nomogram to predict 3- and 5-year overall survival (OS) in training (**A**,**B**) and validation cohorts (**C**,**D**). The X-axis indicated nomogram-predictive survival; the Y-axis suggested actual CSS. A plot with 45-degree line was suggestive of a perfect calibration where predictive possibilities were identical to actual ones. Vertical bars indicated 95% CIs.

**Figure 4:**
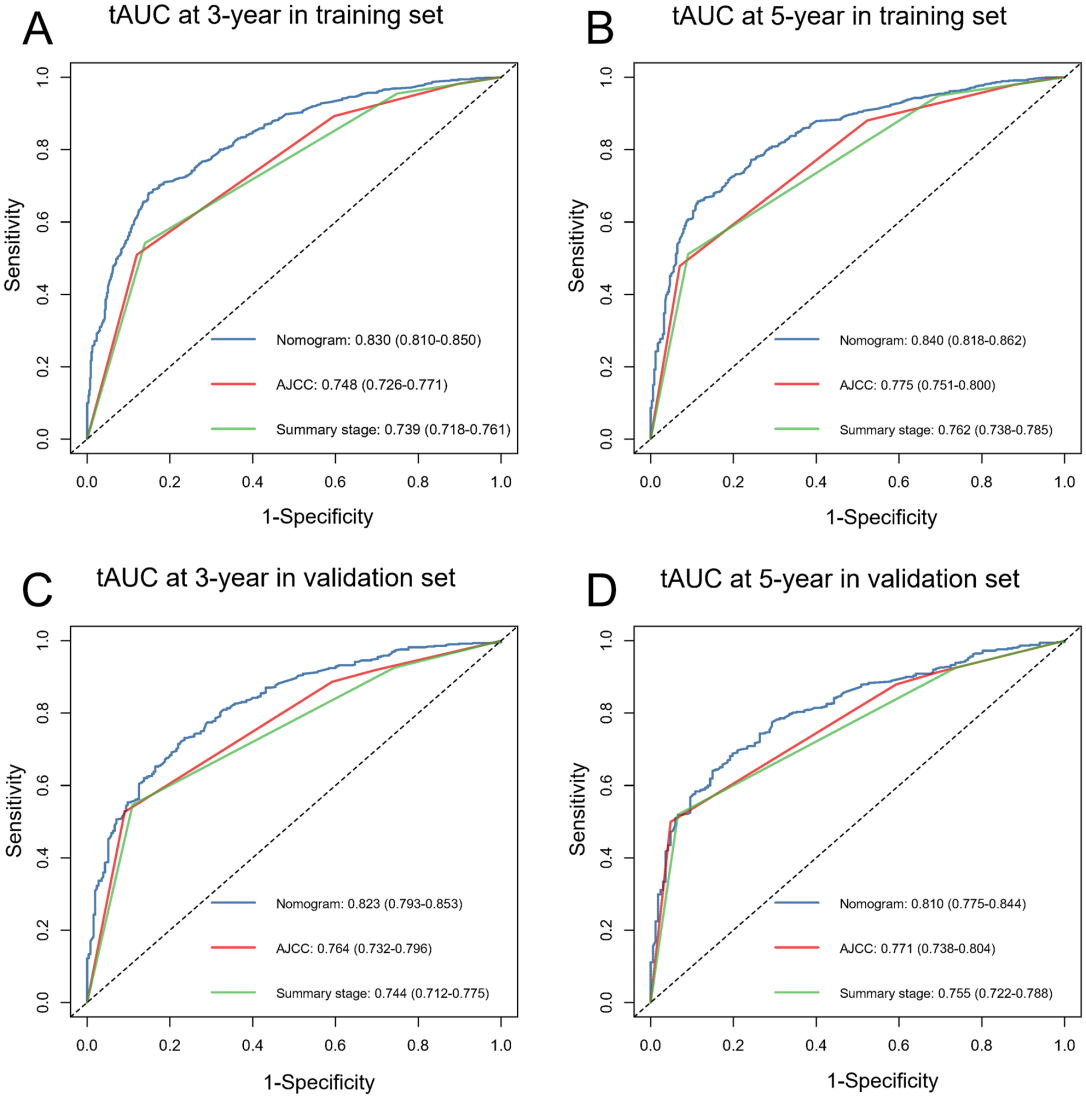
Discriminatory accuracy for predicting OS assessed by receiver operator characteristics (ROC) analysis calculating time independent area under the curves (tAUCs). 3-year (**A**) and 5-year (**B**) in the training cohort; 3-year (**C**) and 5-year (**D**) in the validation cohorts.

The nomogram was validated internally and externally. In the training and validation cohorts, namely internal validation and external validation cohorts, the OS prediction C-indexes in nomogram were respectively 0.743 (95% CI, 0.730–0.755), 0.730 (95% CI, 0.710–0.751). Furthermore, a comparison was made between the discrimination ability of nomogram with the ability of SEER summary stage and TNM 8th staging classification, indicating that in the training as well as validation set (*P* < 0.001), the nomogram is superior to SEER and TNM 8th staging classification, as shown in Table 3. At last, as shown in Fig. 3, both the internal calibration plot and external calibration plot of the nomogram exhibited good consistency between the predictions and practical results based on the nomogram. Figure 4 showed the relevant ROC of the training and validation cohort. In the training cohort, the time independent area under the curves (tAUCs) of 3– and 5– years OS were 0.830 (0.810–0.850) and 0.840 (0.818–0.862). In the validation cohort, the tAUCs of OS for 3- and 5- years were 0.823 (95% CI: 0.793–0.853) and 0.810 (95% CI: 0.775–0.844), respectively, which were all greater than AJCC and SEER summary stage system. Bootstrapping with 1000 resamples in the training set yielded similar discrimination.The 3–year and 5– year tAUCs of the prognostic model in the training set were 0.829(0.812–0.850) and 0.839 (0.820–0.860), respectively.

**Table 3:** C-indexes for the nomogram and other stage systems in patients with colorectal signet ring cell carcinoma.

Classification	Training set		Validation set	
	C-index (95% CI)	*P* value*	C-index (95% CI)	*P* value*
Nomogram	0.743 (0.730, 0.755)		0.730 (0.710, 0.751)	
AJCC 8th stage	0.651(0.637, 0.665)	< 0.001	0.650 (0.627, 0.673)	< 0.001
SEER summary stage	0.645 (0.631, 0.659)	< 0.001	0.639 (0.617, 0.661)	< 0.001

*All are compared with Nomogram, *HR* hazard ratio, *CI* confidence interval.

## Discussion

A prognostic nomogram for 3- and 5-year OS prediction was constructed and validated in our study. We analyzed 2904 colorectal SRCC patients from SEER dataset, followed by constructing a nomogram for 3- and 5-year OS prediction. In addition, internal and external validation of the nomogram demonstrated favorable calibration as well as discrimination. Moreover, our established nomogram showed more potent predictive capacity compared to SEER summary stage or TNM staging systems, which could be readily applied in clinical practice to assist patient counseling as well as individualized therapy.

Some independent factors for predicting prognosis were incorporated into our constructed nomogram. Besides, the survival was also analyzed based on the colorectal SRCC stage, which discovered that early stage patients had better prognosis than those at the advanced stage, and such results conformed to almost every study^27,28^. Ishihara and colleagues discovered that primary location might serve as the independent factor for prognosis prediction^29^. Typically, both tumor stage and primary location were identified as the prognostic factors in the present work. Moreover, this study identified tumor size and pathological grade as the independent prognostic factors for colorectal SRCC.

It is necessary to conduct multidisciplinary treatment of colorectal SRCC, so as to select the best therapeutic strategy, and it should be noted that surgery is significant to treat the localized tumors^30^. As suggested by a population-based study that enrolls 1972 colorectal SRCC patients between 1989 and 2010 to evaluate whether adjuvant chemotherapy is significant, adjuvant chemotherapy can offer survival benefits to stage III colon SRCC patients^31^. Tao Shi and coworkers also discovered that chemotherapy was linked with the superior survival of colorectal SRCC with distant metastasis^32^. Findings in this work also verified that both chemotherapy and surgery played important roles in diagnosing colorectal SRCC. Further, the surgical retrieval of at least 4 regional lymph nodes markedly enhanced patient survival. The above-mentioned factors remarkably impacted colorectal SRCC prognosis. Using our constructed nomogram, patients suffering from diverse tumor differentiation degrees were assigned with different scores and then with diverse survival outcomes, even though they might be at the same TNM stage. Besides, these results explicitly clarified the difference between the prognosis estimated using our constructed nomogram and that estimated by the TNM classification systems, which might explain the better ability of our nomogram in predicting OS than the TNM classification systems.

Previous studies have also explored nomograms in colorectal signet ring cell carcinoma^33,34^. Wang et al. retrospectively evaluated the patient records of mucinous adenocarcinoma and SRCC patients aged ≤ 40 years^34^. A nomogram predicting OS was created for risk quantitation. However, compared with our study, the number of cases enrolled in the previous study was still too small, and only included patients aged ≤ 40 years. Our study may be more comprehensive and practical.

As a statistical method, the nomogram is capable of providing survival possibility by formula calculation^35,36^. Nomogram has been validated to harbor superior predictive capacity in comparison with TNM stage system in certain types of malignant tumors, which is considered as an alternative or even a novel standard^37,38^. In particular, it is proper to use nomogram to handle complicated situations without clinical guidelines. And it is convenient and simple to utilize nomogram for survival prediction. To begin with, in a nomogram, from each clinicopathological parameter, a vertical line is drawn to “scores” line, followed by score addition to give rise to “total scores”. Therefore, certain recommendation could accordingly be offer by clinicians. For instance, operation is suggested in well-differentiated populations in consideration of satisfactory prognosis. On the contrary, palliative chemotherapy is preferred in poorly-differentiated populations in consideration of decreased life expectancy. Thus, the presently established nomogram could help to choose patients with prolonged survival, who might benefit from palliative resection.

Several advantages exist in our research. The detailed clinicopathological data of colorectal SRCC from SEER database ensured that we successfully constructed a precise prognostic nomogram. Moreover, superior discriminative capacity for OS prediction is detected in our nomogram compared to SEER summary stage and TNM stage systems. Additionally, available clinical parameters are used, which is convenient for nomogram application.

Certain limitations should be noted in this population-based study. First of all, selection bias was inevitable due to the retrospective nature. Secondly, prognostic information, including the microsatellite stability/microsatellite instability (MSS/MSI) status, the RAS/BRAF/MSI status, family history, vascular invasion and patient condition, was not available in the SEER database, and future research should focus on these aspects. Thirdly, the convincing external verification was lacking in this work. At last, our constructed nomogram, which might serve as a user-friendly approach for the decision-making of doctors, did not incorporate each prognostic factors or always offer accurate prognosis prediction in clinical practice.

## Conclusion

In conclusion, for patients with colorectal SRCC, we established and validated a nomogram to predict 3-and 5-year OS based on a large, population-based cohort. The nomogram showed excellent performance and could be thought of as a practical tool to predict prognosis. Nevertheless, further mining of the uncertain prognostic parameters for the optimization of nomogram is still needed, which requires in-depth external validation.

## Supplementary Information

Supplementary Figure 1.

Supplementary Figure 2.
