# Supplementary figures and images for: Construction and validation a nomogram to predict overall survival for colorectal signet ring cell carcinoma
Source: Sci Rep. 2021 Feb 9;11:3382. doi: 10.1038/s41598-021-82978-8 (PMC7873243; doi:10.1038/s41598-021-82978-8)

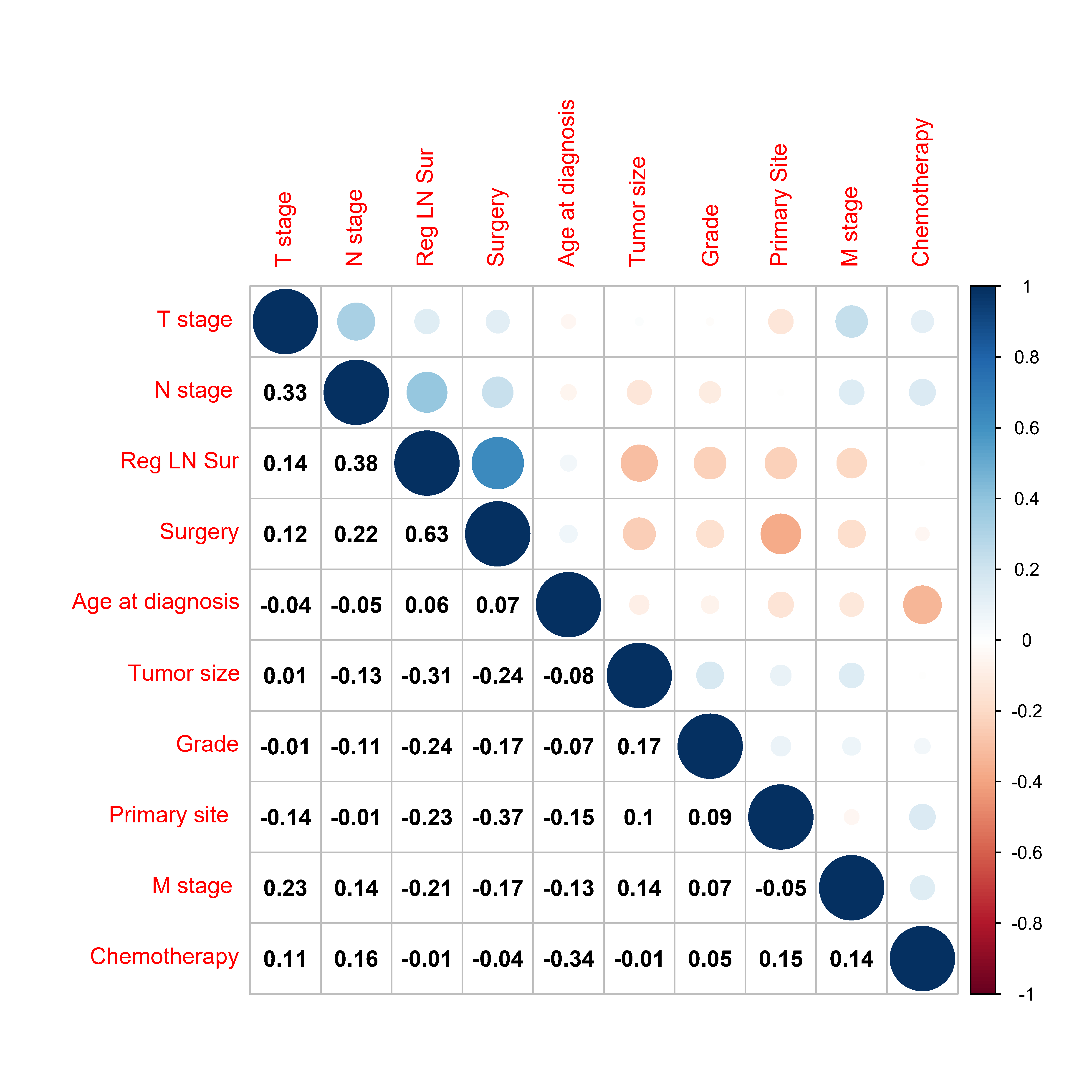

Supplement: Supplementary file 1 — Supplementary Figure 1. [file 41598_2021_82978_MOESM1_ESM.png]

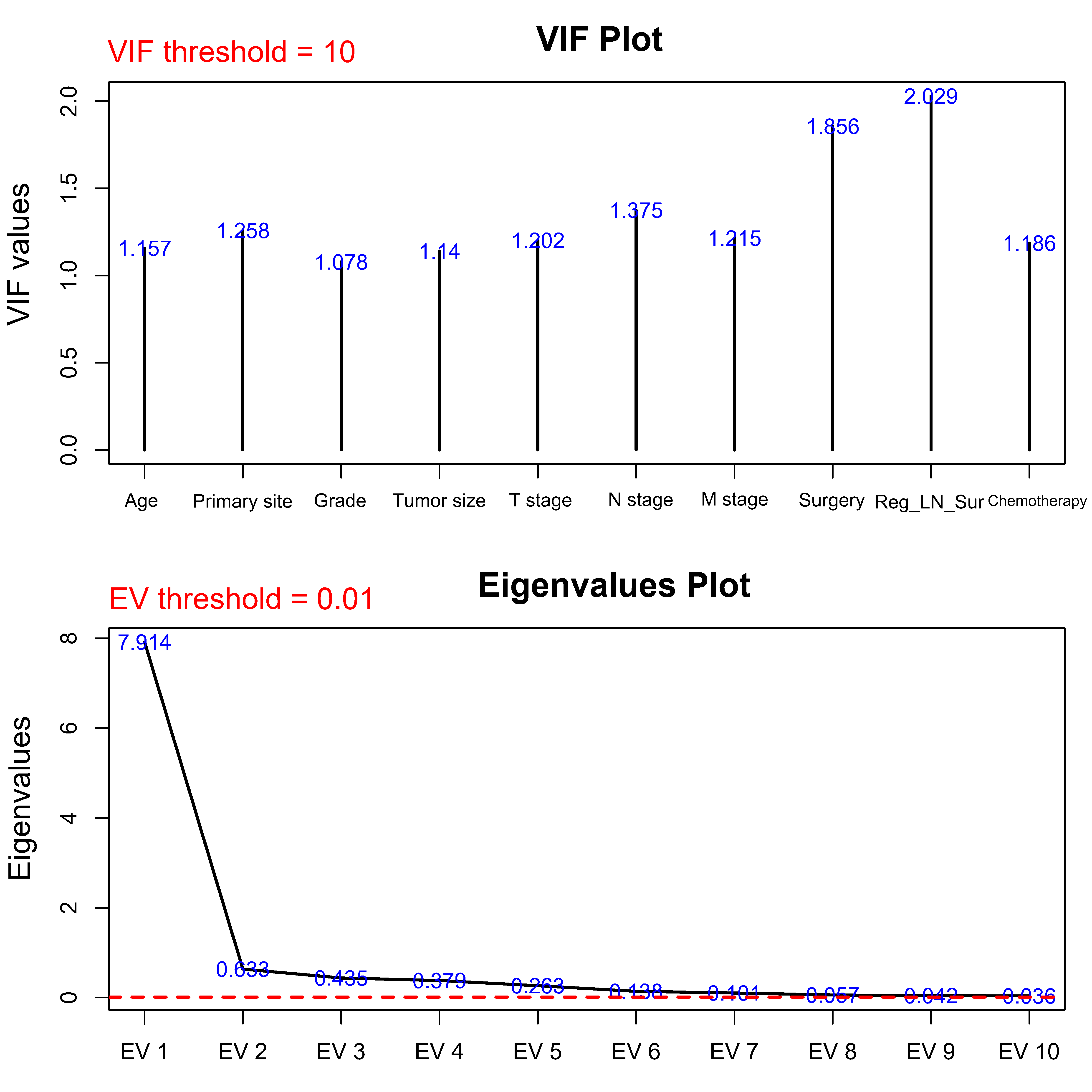

Supplement: Supplementary file 2 — Supplementary Figure 2. [file 41598_2021_82978_MOESM2_ESM.png]
